# Aorto-mitral curtain reconstruction in invasive double-valve endocarditis: mid-term outcomes

**DOI:** 10.3389/fcvm.2023.1154129

**Published:** 2023-05-10

**Authors:** Martin Vobornik, Salifu Timbilla, Jan Gofus, Petr Smolak, James Lago Chek, Marek Pojar, Eva Cermakova, Pavel Zacek, Jan Vojacek

**Affiliations:** ^1^ Department of Cardiac Surgery, Faculty of Medicine and University Hospital in Hradec Kralove, Charles University, Hradec Kralove, Czechia; ^2^ Department of Medical Biophysics, Faculty of Medicine Hradec Kralove, Charles University, Hradec Kralove, Czechia

**Keywords:** infective endocarditis, aorto-mitral continuity, intervalvular fibrosa, commando procedure, hemi-commando procedure

## Abstract

**Background:**

Invasive double-valve endocarditis with structural damage (abscess or perforation) of the aorto-mitral curtain is a relatively rare but fatal diagnosis requiring complex surgical reconstruction. This study presents the short-term and mid-term outcomes from a single center.

**Methods:**

From 2014 to 2021, 20 patients with double-valve endocarditis with structural damage of the aorto-mitral curtain underwent surgical reconstruction (Hemi-Commando procedure *n* = 16 and Commando procedure *n* = 4). Data were obtained retrospectively.

**Results:**

In 13 cases, the procedure was a reoperation. The mean cardiopulmonary bypass time was 239 ± 47 min and the mean cross-clamp time was 186 ± 32 min. Concomitant procedures were tricuspid valve repair in two, coronary revascularization in one, closure of a ventricular septal defect in one and hemiarch (using circulatory arrest) in one patient. Eleven patients (55%) required surgical revision for bleeding. Thirty-day mortality was 30% (6 patients)—3 patients from the Hemi-Commando group (19%) and 3 patients from the Commando group (75%). Overall survival at 1, 3 and 5 years was 60%, 50% and 45% respectively. Reoperation was required by 4 patients. Freedom from reoperation at 1, 3 and 5 years was 86%, 71% and 71% respectively.

**Conclusion:**

Despite the high postoperative morbidity and mortality, complex surgical reconstruction of the aorto-mitral continuity of patients with double-valve endocarditis represents the only real chance for survival. Mid-term outcomes are acceptable, but strict follow-up is required due to the risk of valve failure.

## 1. Introduction

**Figure 1:**
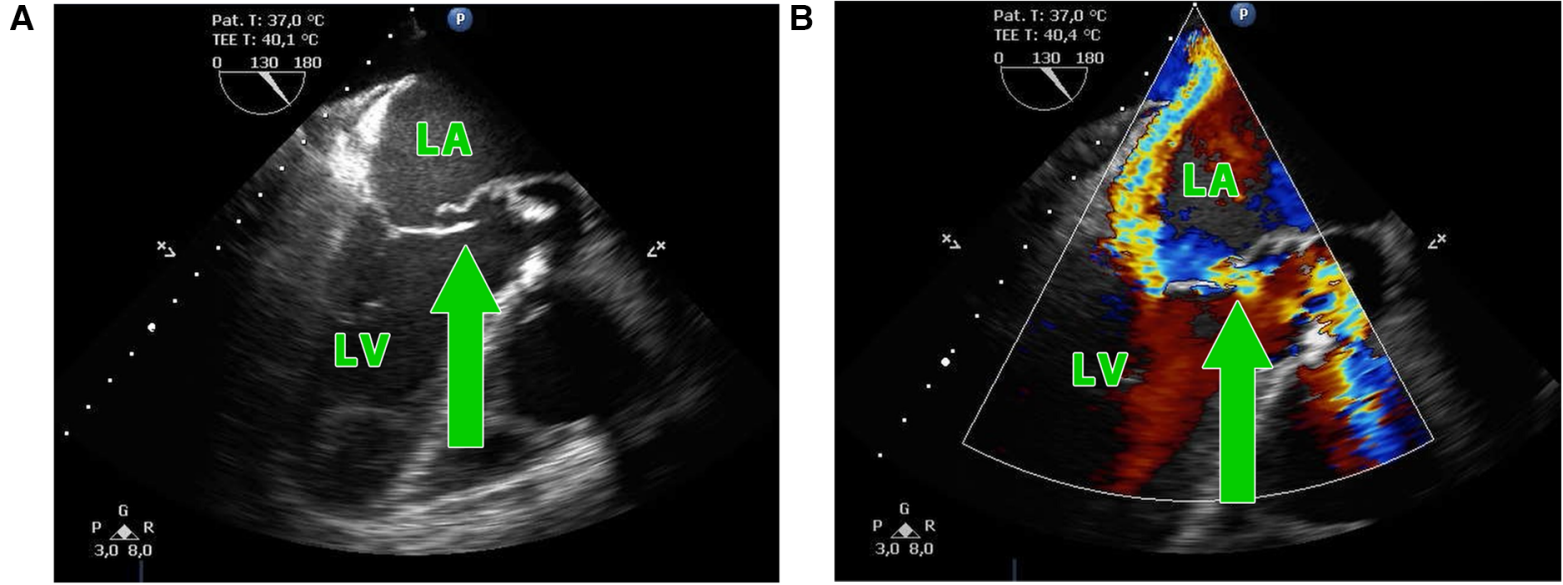
(**A,B**) Transesophageal echocardiography showing the perforation of the aorto-mitral curtain (green arrow) in a patient with double-valve endocarditis; LA, left atrium; LV, left ventricle.

Infective endocarditis (IE) is, despite advanced diagnostics and antibiotic treatment, associated with early mortality of up to 30% and up to half of the patients require surgical intervention (1–3). A relatively rare complication is the involvement of the aorto-mitral curtain (AMC), especially in the cases with invasive double-valve endocarditis in whom disease control was not successful (4). Surgical treatment of the double-valve endocarditis with the destruction of the AMC (Figures 1A,B) necessitates radical debridement of all infected tissue to prevent recurrence of IE (5) and complex high-risk surgical reconstruction of the AMC (6, 7). According to the extent of surgical intervention on the mitral valve, we can distinguish two types of operations—the Commando procedure and the Hemi-Commando procedure. The Commando procedure consists of aortic and mitral valve replacement and the reconstruction of the AMC using a pericardial or Dacron patch (8, 9). In cases where the posterior leaflet of the mitral valve is not affected by the infection and can be saved, the Hemi-Commando procedure may be performed. The surgery includes replacement of the aortic valve and anterior leaflet of the mitral valve and reconstruction of the AMC using a homograft (6, 10, 11). In this study, we present the short-term and mid-term outcomes of the surgical reconstruction of the AMC in patients with invasive double-valve endocarditis from our institution.

## 2. Materials and methods

Between 2014 and 2021, a total of 20 patients with invasive double-valve IE underwent reconstruction of the AMC (Hemi-Commando procedure *n* = 16 and Commando procedure *n* = 4) at our institution. The aim of our study was to compare these two types of high-risk surgical procedures and analyze the risk of death, the risk of the recurrence of IE and the risk of reoperation in these patients. Perioperative and follow-up data were obtained retrospectively from medical records and were approved for use in research as the patients had signed informed consent forms prior to surgery. The study was approved by our Institutional Ethics Committee (Reference number 202301 P07).

The median age of the whole cohort was 62.5 years [IQR 47.8; 65.3] (in the Hemi-Commando group 62.5 years [IQR 49.5; 65.3]; in the Commando group 55.0 years [IQR 44.5; 66.5]) (Table 1). For operative risk evaluation we used the European System for Cardiac Operative Risk Evaluation (EuroSCORE II) (12). Six patients died early after surgery. The remaining 14 patients were followed-up, with a 100% follow-up rate. Most of the patients had follow-up cardiac examinations including echocardiography, every patient had at least 1 examination. The mean follow-up was 36 ± 31 months.

**Table 1:** Preoperative patient characteristics.

Characteristics	Overall cohort (*n* = 20)	Hemi-Commando (*n* = 16)	Commando (*n* = 4)	*p* value
Age (years)	62.5 [47.8; 65.3]	62.5 [49.5; 65.3]	55.0 [44.5; 66.5]	NS
Male sex (*n*)	17	14	3	NS
Body surface area (m^2^)	28.0 [24.4; 31.0]	29.8 [25.7; 31.4]	24.3 [22.4; 26.5]	NS
NYHA (*n*)				NS
I	3	3	0	
II	8	6	2	
III	2	1	1	
IV	7	6	1	
Diabetes mellitus (*n*)	6	5	1	NS
Arterial hypertension (*n*)	10	9	1	NS
Dyslipidemia (*n*)	8	8	0	NS
Baseline ECG (*n*)				NS
Sinus rhythm	8	6	2	
Atrial fibrillation/flutter	9	8	1	
Permanent pacemaker	3	2	1	
Kidney disease (*n*)				NS
None	15	11	4	
Renal failure	3	3	0	
Dialysis	2	2	0	
Kreatinin (μmol/L)	92.5 [70.8; 145.8]	106.0 [74.8; 145.8]	73.0 [59.5; 106.3]	NS
CRP (mg/L)	75.8 [41.6; 96.1]	75.8 [26.3; 96.1]	70.0 [55.9; 89.2]	NS
Aortic regurgitation grade (*n*)				NS
I	7	4	3	
II	2	2	0	
III	1	1	0	
IV	10	9	1	
Mitral regurgitation grade (*n*)				NS
I	4	2	2	
II	5	5	0	
III	3	2	1	
IV	8	7	1	
LVEF (%)	55.0 [40.0; 60.0]	55.0 [47.5; 60.0]	50.0 [40.0; 65.0]	NS
Prior cardiac surgery (*n*)				NS
-0-	7	6	1	
-1-	11	8	3	
-2-	1	1	0	
-3-	1	1	0	
Endocarditis pathology (*n*)				NS
Native valve	7	6	1	
Prosthetic valve	13	10	3	
Structural disability (*n*)				NS
Abscess of AMC	15	11	4	
Fistula in AMC	5	5	0	
EuroSCORE II (%)	16.3 [7.1; 47.9]	16.3 [7.1; 48.4]	19.1 [8.2; 36.4]	NS

The data are presented as median with interquartile range 25th to 75th percentile [median (IQR)] or in absolute numbers (*n*); NYHA, New York Heart Association functional classification; ECG, electrocardiogram; CRP, C-reactive protein; LVEF, left ventricular ejection fraction; AMC, aorto-mitral curtain; EuroSCORE II, European system for cardiac operative risk evaluation.

### 2.1. Surgical technique

**Figure 2:**
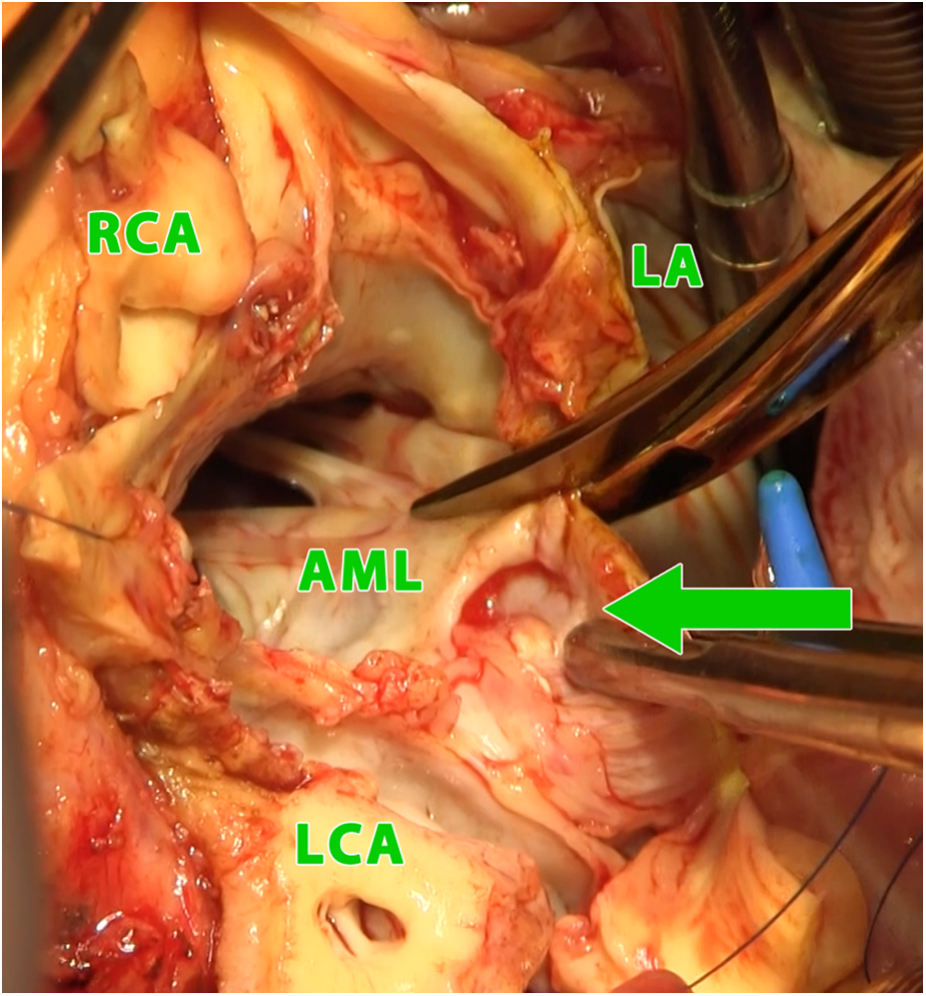
Intraoperative photograph (view into the left ventricular outflow tract and the left and right atrium using combined manouguian-guiraudon approach) showing the perforation of the aorto-mitral curtain (green arrow); LA, left atrium; AML, anterior mitral valve leaflet; RCA, right coronary artery; LCA, left coronary artery.

**Figure 3:**
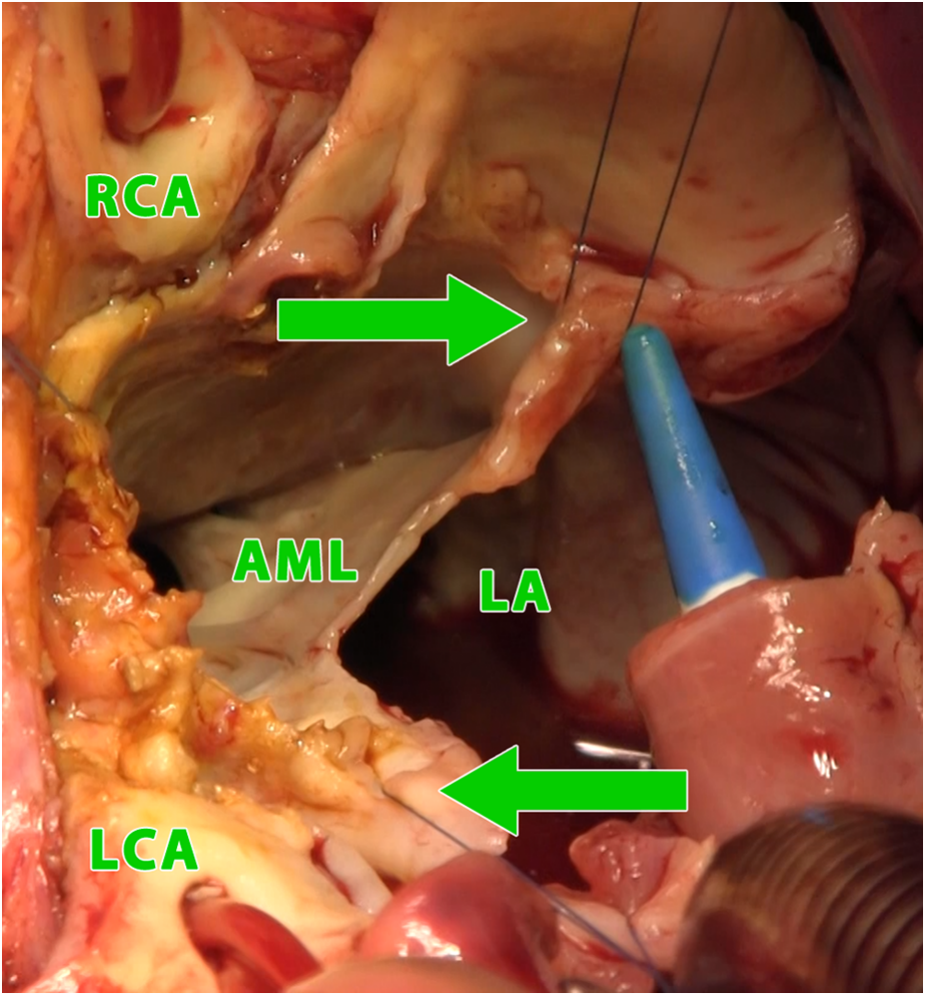
Intraoperative photograph (view into the left ventricular outflow tract and the left and right atrium using combined manouguian-guiraudon approach), showing the status after radical excision of the perforated AMC, stitches in fibrous trigones (green arrows); LA, left atrium; AML, residue of anterior mitral valve leaflet; RCA, right coronary artery; LCA, left coronary artery.

All operations were performed using the median sternotomy approach. Bicaval venous cannulation is mandatory for extensive exposure allowing for a wide opening of the left atrium, and also of the right atrium. In reoperations we preferred combined peripheral cannulation using a venous cannula inserted percutaneously through the jugular vein into the superior vena cava together with peripheral arterial and venous cannulation in the groin. For cardiac arrest we used antegrade delivery of the cardioplegia solution CUSTODIOL® HTK (Essential Pharmaceuticals, LLC). After the heart was arrested, the aorta was transected above the sinotubular junction. The aortic root and the aortic valve were inspected. The infected aortic valve was removed and both coronary buttons were dissected free. For opening the roof of the left atrium we used combined Manouguian-Guiraudon approach (13), which allowed for an excellent exposure and radical removal of all infected tissue (Figures 2, 3).

**Figure 4:**
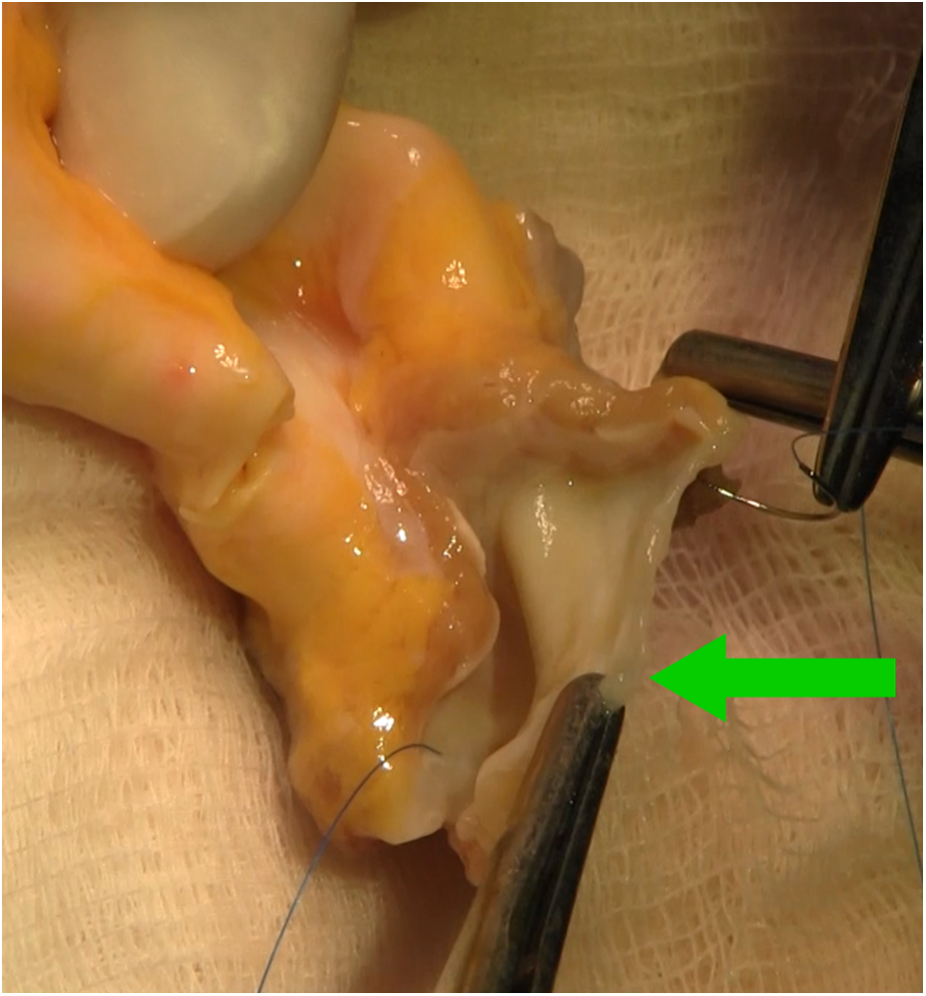
Intraoperative photograph showing aortic homograft with preserved anterior leaflet of the mitral valve (green arrow), stitches in fibrous trigones.

**Figure 5:**
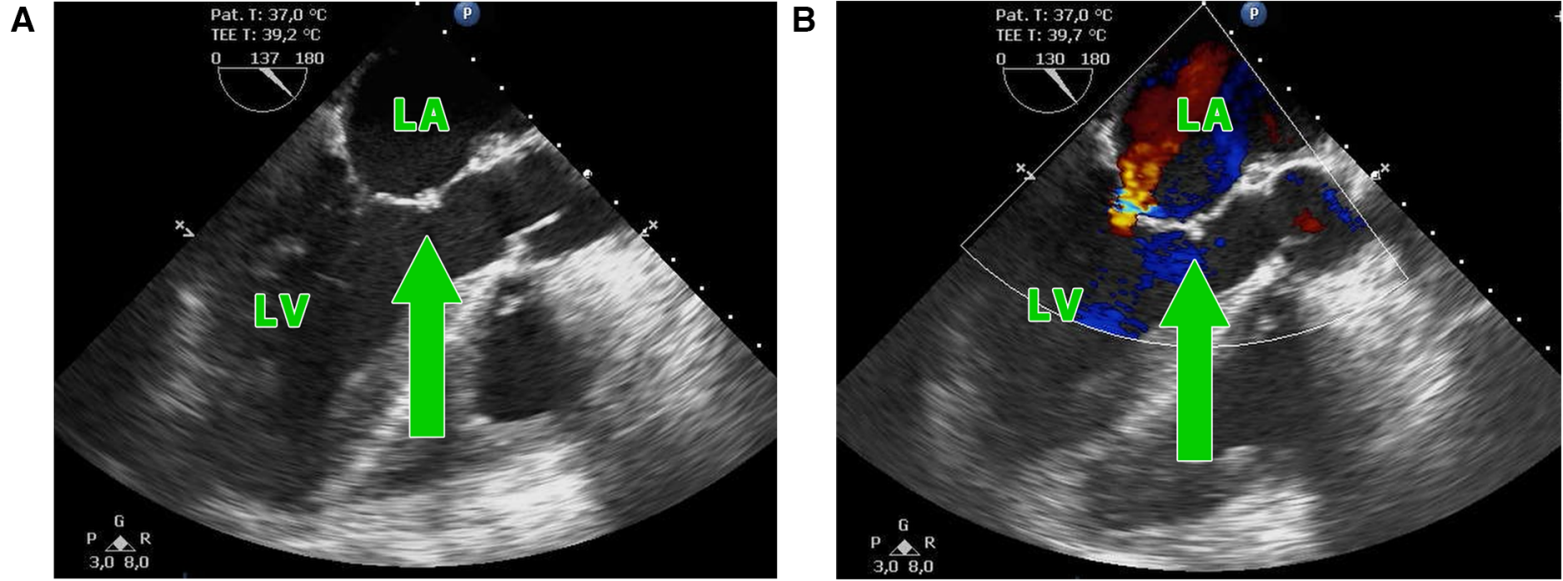
(**A,B**) Postoperative transesophageal echocardiography showing the suture of the anterior mitral leaflet of the homograft with the free edge of the patient's native anterior leaflet (green arrow) with residual mitral insufficiency. LA, left atrium, LV, left ventricle.

The Hemi-Commando procedure could be done only in cases where the posterior leaflet of the mitral valve and free edge of anterior leaflet of the mitral valve with chordae tendineae were not affected by the infection and could be saved. After mitral annuloplasty (using the suture annuloplasty or implantation of an open ring), the AMC was reconstructed with an aortomitral homograft (Figure 4). The homograft was implanted with multiple interrupted non-pledgeted sutures (from the anterior to the posterior trigone). Before the homograft was seated to its position and the sutures tied, the anterior mitral leaflet of the homograft was sutured to the free edge of the patient's native anterior leaflet of mitral valve using 5/0 monofilament running suture (Figures 5A,B). If the Commando procedure was required, the mitral valve was replaced with a prosthesis using multiple interrupted pledgeted sutures, and the AMC was recreated with a pericardial patch or homograft. The roof of the left atrium was closed using the pericardial patch or with the left atrial roof of the homograft. The coronary buttons were reimplanted, the homograft was anastomosed to the ascending aorta, and interatrial septum and the right atriotomy were closed.

### 2.2. Statistical analysis

Data were analysed with NCSS 2021 Statistical Software 2021 (NCSS, LLC. Kaysville, Utah, USA, ncss.com/software/ncss). Qualitative variables are presented in absolute numbers and continuous variables as mean ± SD (standard deviation) or median with 25th to 75th percentile (in case of rejection of normality). Preoperative patient characteristics, operative data and postoperative outcomes were compared using Fisher's exact test. Two-sample *t*-test or Mann-Whitney test were used for continuous variables. Univariate logistic regression were used to evaluate the impact of clinical variables on the 30-day mortality. Level of significance was *α *< 0.05. Survival and freedom from reoperation were estimated with the standard nonparametric Kaplan-Meier curve.

## 3. Results

All 20 patients underwent urgent surgical treatment of double-valve endocarditis involving the AMC. Fifteen patients (75%) had a positive preoperative blood culture test: 3 had Staphylococcus aureus, 3 had Staphylococcus epidermidis, 6 had a pathogen from the Streptococcus group and 3 had other pathogens. Thirteen patients (65%) had a history of prior cardiac surgery. An abscess in the AMC was found in 15 patients (75%), the remaining 5 patients (25%) had a fistula in the AMC. Median EuroSCORE II was 16.3% [IQR 7.1; 47.9] (Table 1).

In 16 patients (80%), we performed the Hemi-Commando procedure with a mean cardiopulmonary bypass time of 236 ± 51 min and a mean cross-clamp time of 184 ± 35 min. Concomitant procedures in the Hemi-Commando group were: tricuspid valve repair in two, coronary revascularization of the right coronary artery in one and hemiarch with hypothermic circulatory arrest in one patient. The remaining 4 patients (20%) required mitral valve replacement (the Commando procedure) with a mean cardiopulmonary bypass time of 251 ± 31 min and a mean cross-clamp time of 197 ± 17 min. The AMC was reconstructed in two cases using a pericardial patch and in another two with a homograft in this group. There was a single concomitant procedure in this group which was the closure of a ventricular septal defect (Table 2).

**Table 2:** Operative data and postoperative outcomes.

Characteristics	Overall cohort (*n* = 20)	Hemi-Commando (*n* = 16)	Commando (*n* = 4)	*p* value
Aortic valve replacement (*n*)				0.0320
Biological prosthesis	2	0	2	
Mechanical prosthesis	0	0	0	
Allograft	18	16	2	
Mitral valve procedure (*n*)				0.0002
Mitral valve repair	16	16	0	
Biological prosthesis	3	0	3	
Mechanical prosthesis	1	0	1	
AMC reconstruction (*n*)				0.0316
Pericardium	2	0	2	
Homograft	18	16	2	
Concomitant procedures (*n*)				NS
TVP	2	2	0	
CABG	1	1	0	
VSD	1	0	1	
Hemiarch	1	1	0	
Cardiopulmonary bypass (min)	231 [213; 259]	223 [209; 259]	238 [231; 257]	NS
Cross-clamp time (min)	186 [163; 210]	178 [160; 210]	202 [193; 207]	NS
Circulatory arrest (*n*)	1	1	0	NS
MCS (*n*)	7	5	2	NS
Delayed chest closure (*n*)	7	6	1	NS
Revision (*n*)	11	9	2	NS
Prolonged ventilation >24 h (*n*)	13	9	4	NS
Atrial fibrillation (*n*)	6	5	1	NS
Pacemaker (*n*)	6	5	1	NS
Stroke (*n*)	2	1	1	NS
Hospital death (*n*)	6	3	3	NS

The data are presented as median with interquartile range 25th to 75th percentile [median (IQR)] or in absolute numbers (*n*); AMC, aorto-mitral curtain; TVP, tricuspid valve plasty; CABG, coronary artery bypass graft; VSD, closing of ventricular septal defect; MCS, mechanical circulatory support.

Short-term mechanical circulatory support was required in 7 patients (35%). Surgical revision for bleeding or delayed chest closure was needed in 11 patients (55%). Six patients (30%) required pacemaker implantation and two patients (10%) had a stroke postoperatively (Table 2). The thirty-day mortality was 30% (6 patients) in the whole cohort—3 patients from the Hemi-Commando group (19%) and 3 patients from the Commando group (75%). We analyzed risk factors of thirty-day mortality using univariate logistic regression (Table 3), but only the Commando procedure was identified as a significant risk factor (*p* = 0.0340).

**Table 3:** Univariate analysis of clinical variables and thirty-day mortality.

Variable	OR	95% CI	*p* value	% Correct classification
Age >60 years	5.00	0.46; 54.51	NS	60
BMI >30 kg/m^2^	3.60	0.48; 27.11	NS	65
Diabetes mellitus	3.67	0.47; 28.40	NS	70
Arterial hypertension	2.67	0.36; 19.71	NS	60
Dyslipidemia	1.80	0.26; 12.50	NS	60
NYHA IV	0.90	0.12; 6.78	NS	45
Tobacco use	3.50	0.37; 32.97	NS	70
Creatinin >105 μmol/L	1.33	0.20; 9.08	NS	55
Dialysis	2.75	0.14; 55.17	NS	65
Arterial hypertension	2.67	0.36; 19.71	NS	60
LVEF <40%	1.20	0.09; 16.44	NS	65
Prior cardiac surgery	1.11	0.15; 8.37	NS	45
Pathologic agent				
Staphylococcus	1.00	0.15; 6.77	NS	30
Streptococcus	2.50	0.35; 18.04	NS	65
Severe aortic regurgitation	0.28	0.04; 2.09	NS	65
Severe mitral regurgitation	6.67	0.61; 73.03	NS	65
Commando procedure	13.00	0.98; 172.95	0.0340	80
Concomitant procedures	0.50	0.04; 5.74	NS	45
MCS	7.33	0.88; 61.33	NS	75

OR, odds ratio; CI, confidence interval; BMI, body mass index; NYHA, New York Heart Association functional classification; LVEF, left ventricular ejection fraction; MCS, mechanical circulatory support.

**Figure 6:**
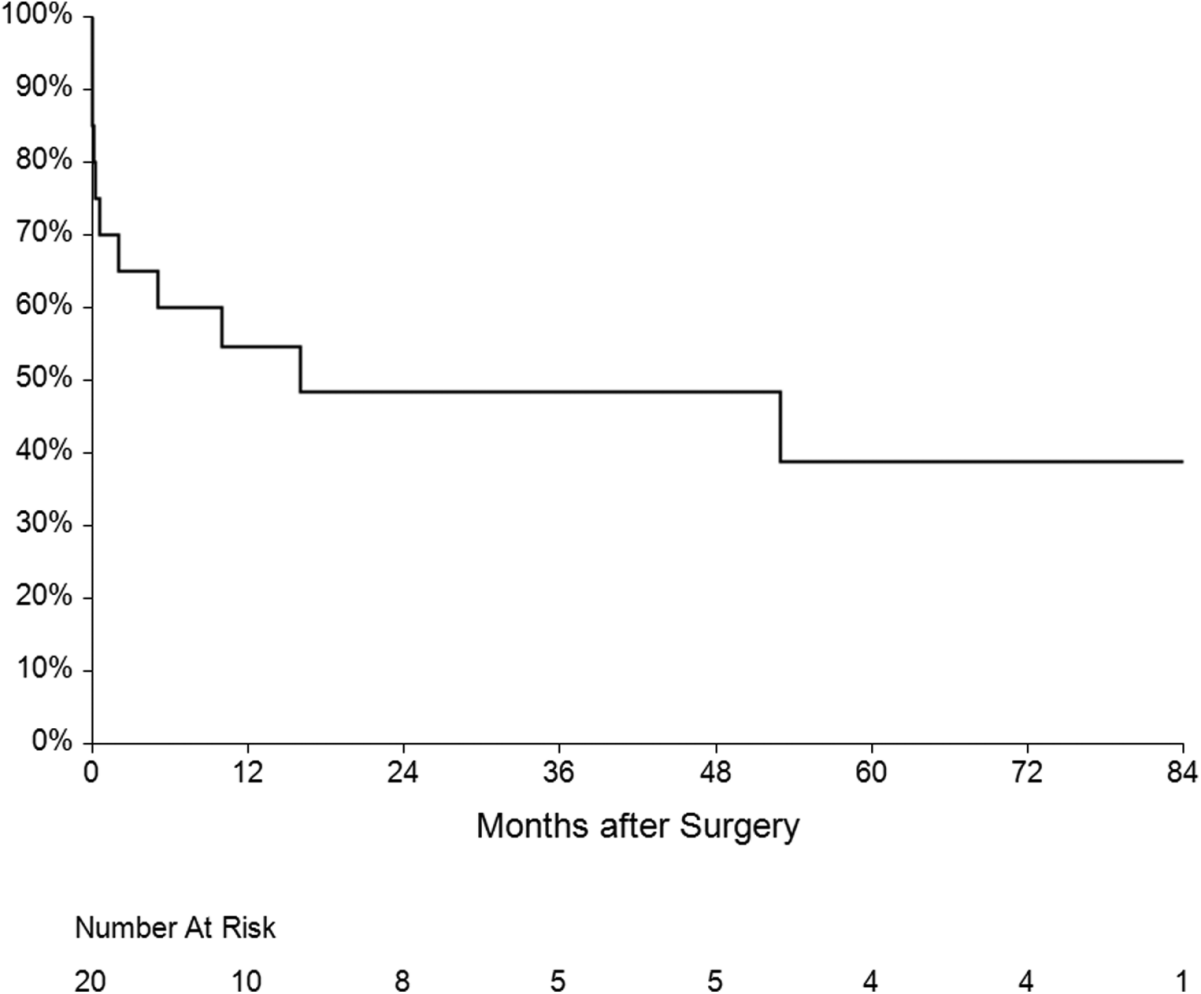
Kaplan-Meier estimate for survival after surgery.

**Figure 7:**
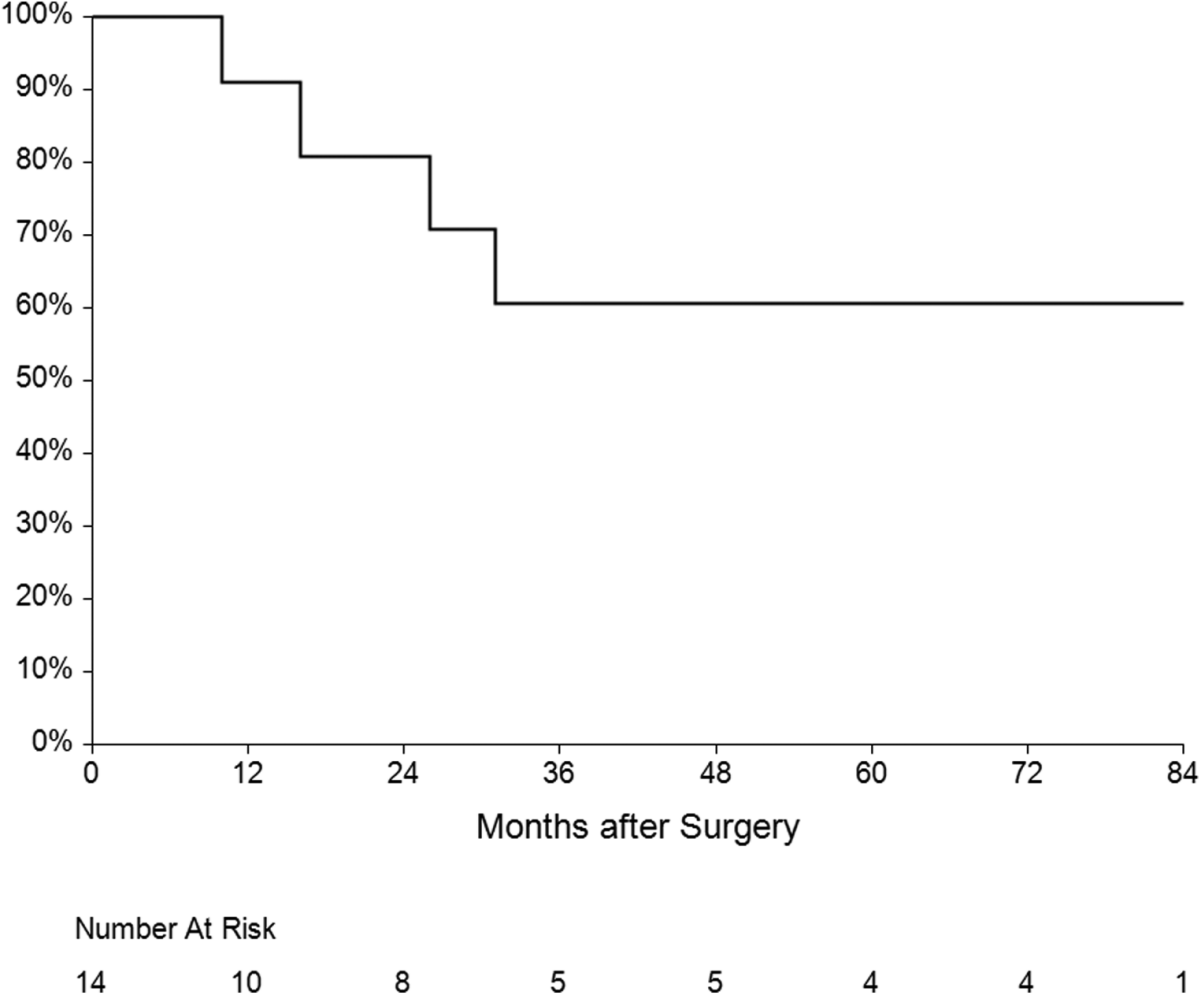
Kaplan-Meier estimate for freedom from reoperation.

The mean follow-up was 36 ± 31 months. One patient had recurrent IE (7.1%) caused by another pathogen 15 months after surgery. Five patients died during the follow-up—three of them from a non-cardiac cause, one died after reoperation for IE and the last one died after reoperation for mitral valve dysfunction. Overall survival at 1, 3 and 5 years was 60%, 50% and 45% respectively (Figure 6). Four patients underwent reoperation—1 patient for IE and 3 patients for mitral valve dysfunction (Table 4). Freedom from reoperation at 1, 3 and 5 years was 86%, 71% and 71% respectively (Figure 7).

**Table 4:** Follow-up data.

Characteristics	Overall cohort (*n* = 14)	Hemi-Commando (*n* = 13)	Commando (*n* = 1)	*p* value
Recurrent IE (*n*)	1	1	0	NS
Reoperation (*n*)	4	3	1	NS
Death after discharge (*n*)	5	4	1	NS
Aortic valve regurgitation (*n*)				NS
None	11	11	0	
Mild	1	1	0	
Modere	1	0	1	
Severe	1	1	0	
Mitral valve regurgitation (*n*)				NS
None	4	4	0	
Mild	3	3	0	
Modere	0	0	0	
Severe	7	6	1	

The data are presented in absolute numbers (*n*); IE, infective endocarditis.

## 4. Discussion

In this paper we present our experience with surgical treatment of advanced IE involving the aorto-mitral curtain using Hemi-Commando or Commando technique. This radical treatment allows us to remove all the infected tissue and provide the patient with a chance to survive an otherwise lethal condition. The life-saving procedure is associated with a high risk of bleeding, need of mechanical circulatory support, prolonged stay in the intensive care unit and a relatively high short-term mortality. On the other hand, the surgery is very effective in terms of eradication of the triggering pathogen as recorded in our series. After curing the initial disease, the surgery carries a long-term postoperative risk of mitral valve failure despite the very delicate anastomosis of the homograft mitral valve with patient's native anterior mitral valve leaflet.

Apart from AMC reconstruction in cases of invasive double-valve IE with the destruction of the AMC (8, 10, 14), this procedure can also be performed in patients indicated for aortic and mitral valve replacement with a small annulus requiring expansion as a prevention of patient-prosthesis mismatch (15), in patients with extreme AMC calcifications (16–18) or during repeat reoperations when there is not enough high-quality tissue (9). The generally low numbers of these procedures performed is probably one of the main reason why there are few papers in the literature describing the results of AMC surgery (7–11, 19–23). Publications describing only the patients with destructive infective endocarditis are even fewer (only 5), ranging from 14 to 138 patient cohorts (median 37) (7, 10, 21–23). These are similar sets of patients, in which approximately three quarters of patients had prior cardiac surgery, except for the work of Jiang et al. (23), where only 1 patient (7.1%) had a prior operation.

There are 3 main questions in AMC surgery for double-valve IE, which are: what is the risk of death, what is the risk of the recurrence of IE and what is the risk of reoperation. We calculate the risk of surgery preoperatively using the EuroSCORE II risk stratification model, however this has its limitations and the predicted risk in these patients often does not correspond to the actual risk of surgery. The hospital mortality was 30% in the overall cohort, similar to the mortality rates from other published papers (7%–37%) (7, 10, 21–23). In the Commando group, hospital mortality was even higher, 75% (3 out of 4 patients died), which may be inaccurate due to the small sample size. On the other hand, these were patients with more severe IE also affecting the posterior leaflet of the mitral valve and so with a worse intraoperative finding. With regard to these outcomes, the decision to operate on a patient with extensive damage of the posterior mitral valve leaflet necessitating a Commando procedure must be weighed against a very high mortality risk and should be reserved to patients with good biological reserves. Five patients died after discharge, the overall survival at 1, 3 and 5 years was 60%, 50% and 45% respectively, which is similar to the survival rates reported in comparable papers (7, 21, 22).

The second important question is the risk of the recurrence of IE. Out of our group of patients, only one patient (7.1%) developed a recurrence of IE 15 months after their initial surgery. This recurrence was caused by a different pathogen than the one that caused the initial IE. The low recurrence of IE is thanks to the radical debridement performed during surgery with the removal of all infected tissue. These results are comparable to those of other papers where the incidence of recurrence of IE is between 0% and 26% (7, 21, 22).

The third serious question is the risk of reoperation in patients undergoing double-valve surgery with the reconstruction of the AMC. During follow-up, 4 patients underwent reoperation—1 patient for IE and 3 patients for mitral valve dysfunction (one of them from the Commando group). Freedom from reoperation at 1, 3 and 5 years was 86%, 71% and 71% respectively. Only Navia et al. (7), Davierwala et al. (22) and Tomšič et al. (21) published 5 years of follow-up. In these studies, the freedom from reoperation was 83%–87% at 1 year and 51%–85% at 5 years, which is similar to our results.

### 4.1. Limitations

Several limitations of our study have to be acknowledged. This is a single center retrospective study with a relatively small cohort of patients which influences the statistical results. However, this cohort of patients was operated on by one experienced surgeon, thus eliminating intersurgeon variability and limiting a wider application of the outcomes.

## 5. Conclusion

Invasive double-valve endocarditis with the destruction of the AMC is a life-threatening condition with almost 100% mortality as intensive antibiotic treatment alone cannot treat the disease. Despite the relatively high postoperative morbidity and mortality, complex surgical intervention to reconstruct the AMC is necessary and must be performed urgently. If the perioperative finding allows the posterior leaflet of mitral valve to be preserved, the Hemi-Commando procedure is the preferred option, but only radical debridement provides a real chance of successful treatment. During follow-up the incidence of recurrent infective endocarditis was low but strict follow-up is required due to the risk of valve failure.

## Data Availability

The original contributions presented in the study are included in the article, further inquiries can be directed to the corresponding author.
